# Clinical Characteristics of Spontaneous and Recurrent Pneumomediastinum and Risk Factors of Recurrence

**DOI:** 10.1155/emmi/9027140

**Published:** 2026-10-26

**Authors:** Chengbin Huang, Zhiyang Xu, Jianyang Wu, Junkai Xiong, Zhizhou Chen, Sheng Ouyang, Jianxin Xu, Jun Guan

**Affiliations:** ^1^ The Graduate School of Fujian Medical University Fuzhou China fjmu.edu.cn; ^2^ Department of Thoracic Surgery The First Hospital of Putian City Putian China

**Keywords:** clinical features, recurrence, risk factors, spontaneous pneumomediastinum

## Abstract

**Background:**

Spontaneous pneumomediastinum (SPM) is rare, and most existing knowledge comes from small case series. Although usually benign, SPM often leads to hospital admission and substantial healthcare resource use. This study aimed to characterize SPM and recurrent spontaneous pneumomediastinum (RSPM) in a large cohort and to explore factors associated with recurrence.

**Methods:**

Patients with SPM hospitalized at Putian First Hospital between September 2011 and September 2023 were included. Clinical data were extracted from electronic medical records. Follow‐up was performed to identify RSPM. Patients were divided into nonrecurrence and recurrence groups, and risk factors for recurrence were evaluated using univariate logistic regression.

**Results:**

A total of 135 patients with SPM were included. Most patients were male (78.1%), with a median age of 20 years (interquartile range [IQR]: 17–32). A bimodal age distribution was observed, with peaks at 15–19 and 50–54 years. The most common symptom was chest pain (51.9%), and 54.0% of the patients had no identifiable trigger. Among the 9 patients with RSPM (6.7%), median age at recurrence was 25 years (IQR: 16–56) and median interval to recurrence was 6.5 months (range: 1–16.5). Dyspnea was the most common symptom at recurrence (66.7%), and 44.4% of the patients showed greater clinical and radiological severity than at initial presentation. Univariate analysis suggested that a history of bronchitis was associated with RSPM (OR: 11.71, 95% CI: 1.68–81.89); other potential factors included silicosis, pneumonia, and upper respiratory infection as a trigger.

**Conclusion:**

SPM predominantly affects young males and generally follows a benign course. Although recurrence is uncommon (6.7%), it may be more severe than the initial episode in nearly half of the cases. A history of bronchitis may be associated with recurrence, but this finding is exploratory. Age‐specific preventive strategies and careful exclusion of secondary pneumomediastinum are essential.

## 1. Background

Spontaneous pneumomediastinum (SPM) is a rare disease with an overall incidence of 1/12,000 and 1/30,000 [1], and its incidence in all adult inpatients is between 0.001% and 0.01% [2]. Because of its rarity, most existing knowledge comes from case reports or small case series, and large‐cohort studies are scarce. The clinical diversity of SPM often leads to misdiagnosis or delayed diagnosis, and risk factors for recurrence have not been systematically investigated.

SPM is defined as the occurrence of free gas in the mediastinum without any obvious cause [3]. However, the definition of “no obvious cause” varies across studies. The strictest definition excludes any identifiable precipitating event, including underlying lung diseases such as asthma or chronic obstructive pulmonary disease (COPD). In contrast, a broader, more clinically pragmatic definition, which excludes only external or iatrogenic causes (e.g., chest trauma, mechanical ventilation, esophageal rupture, or thoracic surgery), is widely adopted in large case series [4–6]. Under this broader definition, patients with pre‐existing respiratory conditions are classified as having SPM as long as there is no direct external or procedural insult. In the present study, we followed this broader definition to reflect real‐world clinical practice and to maintain comparability with most recent SPM cohorts.

Pneumomediastinum should be distinguished from secondary pneumomediastinum, which results from identifiable structural lesions such as esophageal rupture (Boerhaave syndrome), tracheobronchial disruption, chest trauma, or iatrogenic procedures [7, 8]. Secondary cases often require urgent surgical or endoscopic intervention and carry substantially higher morbidity and mortality [9]. The distinction between SPM and secondary pneumomediastinum is clinically critical but can be challenging, particularly for subtle esophageal perforations or small airway tears, which may initially present similarly but demand completely different management [10].

The pathophysiological mechanism of SPM is the Macklin effect: a sudden rise in intra‐alveolar pressure (e.g., from coughing, vomiting, asthma attack, or physical exertion) causes alveolar rupture, and air dissects along pulmonary vascular sheaths toward the mediastinum [11]. Recognized triggers include acute asthma exacerbation, upper respiratory tract infection, strenuous exercise, diabetic ketoacidosis (DKA), and vomiting [12–16]. The clinical presentation is highly variable, with chest pain and dyspnea being most common; neck pain, subcutaneous emphysema, and Hamman’s sign may also occur, though the latter is inconsistently detected [17].

Although SPM typically follows a benign, self‐limiting course, its clinical impact is not negligible. Patients often present to emergency departments with acute symptoms that raise concern for life‐threatening conditions, prompting extensive diagnostic workups (computed tomography, esophagography, or even bronchoscopy) and unnecessary hospital admissions. In a recent study, most adolescents and young adults with SPM were admitted, and a large proportion underwent CT imaging that may not have been strictly necessary [18]. Hospital length of stay for SPM ranges from 1 to 9 days, contributing substantially to healthcare resource utilization and costs [18, 19].

Recurrent spontaneous pneumomediastinum (RSPM) refers to one or more additional episodes of pneumomediastinum occurring after complete resolution of the initial SPM. It is rare, with reported recurrence rates of 0%–4.5% [20–25]. Some authors have suggested that recurrence does not require special monitoring or treatment because recurrent episodes are often mild [4, 18]. However, evidence supporting this view is limited, as only a few small case series have specifically examined RSPM. Consequently, the true clinical characteristics of RSPM and the factors associated with recurrence remain poorly defined.

To address these gaps, the present study was designed with three main objectives: (1) to describe the clinical characteristics of SPM in a large single‐center cohort; (2) to compare disease presentation between different age groups based on a bimodal age distribution observed in our data; and (3) to explore the clinical features of RSPM and potential risk factors for recurrence, with particular attention to the role of underlying lung diseases.

## 2. Methods

### 2.1. Study Population

The patient identification and selection process is illustrated in Figure 1. Using International Classification of Diseases (ICD)‐9 (518.1) and ICD‐10 codes (J98.2), we screened 405 patients diagnosed with pneumomediastinum at our center between September 2011 and September 2023. After applying the inclusion and exclusion criteria and excluding cases with incomplete clinical data, 135 patients with SPM (including 9 with RSPM) were included in the final analysis. The inclusion criteria were as follows: (1) clinical manifestations consistent with SPM, such as chest pain, dyspnea, and subcutaneous emphysema; (2) no clear cause of induction; (3) free gas around the mediastinum confirmed by chest radiography or high‐resolution computed tomography (HRCT); and (4) patients were aged > 13 years at the time of diagnosis. The exclusion criteria were as follows: (1) a clear cause of pneumomediastinum, such as esophageal rupture, bronchial rupture, pneumomediastinum after thoracic surgery, chest trauma, and prior iatrogenic procedures such as tracheal intubation and closed thoracic drainage and (2) a history of gas‐forming bacterial infection.

**FIGURE 1:**
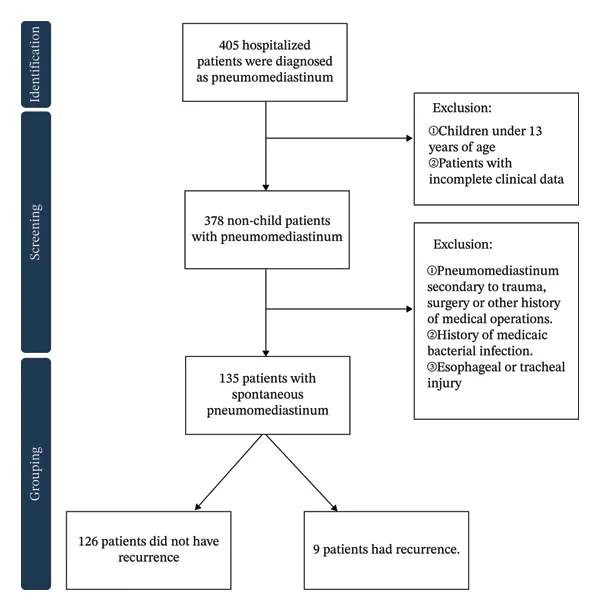
Flowchart of patient selection.

### 2.2. Ethics Approval and Consent to Participate

This retrospective study was reviewed and approved by the Ethics Committee of The First Hospital of Putian City (approval number: 2026‐162, dated August 20, 2026). As the study involved only the retrospective review of anonymized clinical data without any intervention in patient management, the requirement for informed consent was waived. The study adhered to the principles of the Declaration of Helsinki. The ethics approval was obtained retrospectively after data collection was completed, which is permitted for retrospective studies using anonymized data.

### 2.3. Clinical Records

The medical records of all patients who met the criteria were reviewed, and clinical information was obtained from the electronic medical records, including demographics, length of hospital stay (LOS), past medical history, main symptoms, complications (such as subcutaneous emphysema or pneumothorax), predisposing factors, smoking history, laboratory examination, imaging, treatment plan, and clinical symptom outcomes. Among them, the trigger factor was defined as a recent (within 1 week) activity or an event described in the medical record as the potential cause of SPM. Based on the patients’ complaints, the treatment outcomes of clinical symptoms were divided into improvement and aggravation. According to the evaluation of radiologists and clinicians, the absorption of free gas on imaging after treatment was divided into complete absorption, partial absorption, and increase.

All chest imaging studies (chest radiography and HRCT) were independently reviewed by two board‐certified radiologists who were blinded to patients’ clinical outcomes. Discrepancies were resolved by consensus, with a third senior radiologist consulted if needed. The presence and extent of pneumomediastinum and subcutaneous emphysema were assessed using a standardized proforma based on the distribution of free air on axial CT images. The chest aspect ratio was measured on the axial slice at the level of the second rib‐sternum junction.

No patient in this cohort had a documented COVID‐19 or influenza infection at the time of SPM diagnosis; however, viral testing was not performed universally, and the presence of undetected asymptomatic infections cannot be ruled out.

### 2.4. Follow‐Up and Recurrence Definition

Patients were followed through review of inpatient and outpatient (OP) medical records until September 30, 2023. For patients diagnosed before September 1, 2020, the potential follow‐up duration was up to 3 years; for those diagnosed later, the follow‐up duration varied depending on the date of diagnosis. The exact follow‐up time for each nonrecurrent patient was not systematically recorded, which is a limitation of this retrospective study. However, among the 9 patients who experienced recurrence, the median interval from initial SPM to recurrence was 6.5 months (range, 1–16.5 months). Because the follow‐up was passive (based on routine clinical visits rather than scheduled surveillance), mild or asymptomatic recurrences that did not prompt medical attention may have been missed. Recurrence was defined as new‐onset mediastinal free air confirmed by chest imaging after complete resolution of the initial episode.

### 2.5. Statistical Analysis

Continuous variables were first tested for normality using the Shapiro–Wilk test. Normally distributed variables were expressed as mean ± SD and compared using one‐way ANOVA; nonnormally distributed variables were expressed as median (interquartile range [IQR]) and compared using the Kruskal–Wallis H test or Mann–Whitney *U* test as appropriate. Categorical variables were compared using the chi‐square test or Fisher’s exact test. Due to the small number of recurrent events (*n* = 9), only univariate logistic regression was performed to explore potential factors associated with SPM recurrence; multivariate analysis was not conducted. Results are presented as odds ratios (ORs) with 95% confidence intervals (CI). A two‐sided *p* < 0.05 was considered statistically significant. All statistical analyses were performed using SPSS software (Version 27.0; IBM Corp., Armonk, NY, USA).

## 3. Results

The final cohort included 135 patients with SPM, among whom 9 (6.7%) had at least one recurrence. Baseline demographic and clinical characteristics are presented in Table 1. Males accounted for the majority of subjects, with a median age of 20 years. Smoking history was identified in 16.3% of the patients, while nearly one‐quarter (23%) had at least one underlying respiratory comorbidity, with asthma as the most prevalent. Diabetes mellitus was documented in 5.9% of the participants.

**TABLE 1:** Baseline characteristics of the study patients.

Characteristic	Value
No. of patients, *n*	135
Age (years), median (IQR)	20.00 (16.00,35.00)
Hospital day, median (IQR)	5.00 (3.00, 8.00)
Admitted to hospital due to SPM	
Yes	86 (63.7%)
No	49 (36.3%)
Onset time, *n* (%)	
Acute onset	108 (80.0)
Slow onset	27 (20.0)
Sex, *n* (%)	
Male	106 (78.5)
Female	29 (21.5)
Smoking history, *n* (%)	
Smoker	22 (16.3)
Nonsmoker	113 (83.7)
MH, *n* (%)	
Asthma	14 (10.4)
Silicosis	7 (5.2)
PTB	5 (3.7)
Bronchitis	5 (3.7)
COPD	8 (5.9)
Diabetes	8 (5.9)
Complication, *n* (%)	
Pneumonia	40 (29.6)
Pneumothorax	23 (17.0)
Emphysema/PB	23 (17.0)
Trigger factor, *n* (%)	
Asthma	7 (5.2)
Upper respiratory infection	22 (16.3)
Vomitting	5 (3.7)
DKA	6 (4.4)
Physical exertion	10 (7.4)
Without trigger	72 (53.3)
Symptoms, *n* (%)	
Chest pain	70 (51.9)
Neck pain	16 (11.9)
Chest tightness	58 (43.0)
Dyspnea	67 (49.6)
Cough	37 (27.4)
Nausea	13 (9.6)
Radiological data	
Chest aspect ratio, mean (SD)	0.66 (0.07)
Subcutaneous emphysema, *n* (%)	40 (29.6)
Chest	15 (11.1)
Neck	34 (25.2)
Shoulder	9 (6.7)
Laboratory data	
Maximum WBC count (10^9^/L), median (IQR)	9.73 (7.32, 12.35)
Neutrophil (%), median (IQR)	72.28 (64.63, 80.53)
Albumin (g/L), mean (SD)	43.40 (5.39)
Uric acid (μmol/L), median (IQR)	385.00 (303.00, 464.00)
Total cholesterol (mmol/L), median (IQR)	4.00 (3.41, 4.70)

*Note:* IQR, interquartile range; PTB, pulmonary tuberculosis; DKA, diabetic ketoacidosis.

Abbreviations: COPD, chronic obstructive pulmonary disease; MH, medical history; PB, pulmonary bulla; SD, standard deviation; WBC, white blood cell.

Chest pain and dyspnea were the most frequent presenting symptoms. Notably, more than half of the patients had no identifiable trigger; among those with a recognized trigger, upper respiratory tract infection was the most common, followed by physical exertion and asthma. All patients underwent imaging evaluation prior to admission. Subcutaneous emphysema was observed in approximately 30% of the patients, most frequently involving the neck, while Hamman’s sign was not documented in any case. The mean chest aspect ratio was 0.66 ± 0.07.

The median hospital stay was 5 days (IQR: 3–9). Of the 135 patients, 63.7% were admitted primarily for SPM, whereas the remainder was hospitalized for other conditions and received concurrent monitoring for SPM. Pulmonary complications included pneumonia (29.6%), pneumothorax (17.0%), and pulmonary bullae or emphysema (17.0%). Except for two patients who required closed thoracic drainage for pneumothorax, all others received conservative management with oxygen inhalation, analgesia, and targeted treatment for underlying comorbidities (e.g., bronchodilators for asthma/COPD and insulin and fluid therapy for DKA).

Laboratory findings showed a median white blood cell count of 9.73 × 10^9^/L, a median neutrophil proportion of 72.3%, and a mean serum albumin level of 43.4 g/L.

At discharge, most patients showed symptomatic improvement; seven patients were discharged or transferred with persistent symptoms. Follow‐up imaging was available in 98 patients (72.5%): complete resolution was observed in 28 cases (20.7%), partial resolution in 69 cases (51.1%), and progressive disease in one case (0.7%). No deaths or serious complications occurred.

As shown in Figure 2, the age distribution of SPM exhibited a bimodal pattern, with peaks at 15–19 years and 50–54 years. The cutoffs of < 18 years and ≥ 50 years were chosen not only based on these visual peaks but also because they represent physiologically distinct populations: adolescents with typically healthy lungs and older adults with age‐related pulmonary changes and greater comorbidity burden. Based on this distribution, we compared clinical characteristics between patients at the first peak (< 18 years, *n* = 47) and those at the second peak (≥ 50 years, *n* = 24). The results are summarized in Table 2.

**FIGURE 2:**
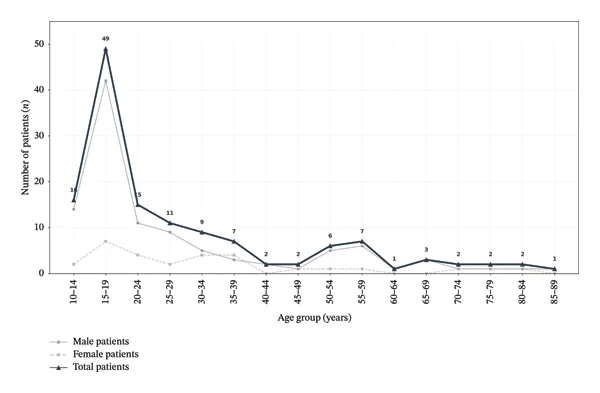
Age distribution of patients with spontaneous pneumomediastinum (SPM) by 5‐year age intervals.

**TABLE 2:** Comparison of clinical characteristics between young patients and older patients.

Characteristic	< 18 years (*n* = 47)	≥ 50 years (*n* = 24)	*p* value
Sex, *n* (%)			0.272
Male	43 (91.5)	19 (79.2)	
Female	4 (8.5)	5 (20.8)	
Hospital day, mean (SD)	4.47 (2.78)	10.17 (6.98)	< 0.001
Smoking (%)	0 (0.0)	14 (58.3)	< 0.001
MH, *n* (%)			
COPD (%)	0 (0.0)	7 (29.2)	0.001
Silicosis (%)	0 (0.0)	5 (20.8)	0.006
Complication, *n* (%)			
Pneumonia	3 (6.4)	17 (70.8)	< 0.001
Pneumothorax	7 (14.9)	8 (33.3)	0.135
Trigger factor, *n* (%)			
Acute exacerbation of pneumonia	3 (6.4)	10 (41.7)	0.001
Acute exacerbation of COPD	0 (0.0)	5 (20.8)	0.006
Symptoms, *n* (%)			
Chest pain	32 (68.1)	6 (25.0)	0.001
Neck pain	10 (21.3)	0 (0.0)	0.038
Dyspnea	14 (29.8)	22 (91.7)	< 0.001
Cough	4 (8.5)	16 (66.7)	< 0.001
Laboratory data			
Neutrophil (%), mean (SD)	66.50 (12.16)	77.63 (10.41)	< 0.001
Uric acid (μmol/L), mean (SD)	431.91 (103.61)	319.57 (209.63)	0.004

*Note:* The < 18 years group includes patients aged 13–17 years, as children under 13 were excluded from the study. IQR, interquartile range.

Abbreviations: PB, pulmonary bulla; SD, standard deviation; WBC, white blood cell.

Compared with the adolescent group (< 18 years), the older group (≥ 50 years) had significantly longer hospital stays (mean 10.17 vs. 4.47 days, *p* < 0.001) and higher smoking rates (58.3% vs. 0%, *p* < 0.001). Underlying lung diseases such as COPD (29.2% vs. 0%, *p* = 0.001) and silicosis (20.8% vs. 0%, *p* = 0.006) were markedly more frequent in the older group, as were pulmonary complications including pneumonia (70.8% vs. 6.4%, *p* < 0.001). Regarding symptoms, chest pain (68.1% vs. 25.0%, *p* = 0.001) and neck pain (21.3% vs. 0%, *p* = 0.038) predominated in the adolescent group, whereas dyspnea (91.7% vs. 29.8%, *p* < 0.001) and cough (66.7% vs. 8.5%, *p* < 0.001) were more common in the older group. Laboratory findings showed higher neutrophil percentages (77.63% vs. 66.50%, *p* < 0.001) but lower uric acid levels (319.57 vs. 431.91 μmol/L, *p* = 0.004) in the older group.

Clinical characteristics of the nine patients with RSPM are summarized in Table 3. The majority were male (77.8%), with a median age of 25 years (IQR, 16–56). Five patients (55.6%) had pre‐existing lung‐related diseases (silicosis, bronchitis, tuberculosis, or asthma). The median interval between the first SPM and recurrence was 6.5 months (range, 1–16.5 months).

**TABLE 3:** Clinical features of 9 patients with recurrent spontaneous pneumomediastinum (RSPM).

Case	Age (*y*)	Sex	BMI (kg/m^2^)	Medical history	Smoking	Interval (months)	Initial episode				Recurrence				Comparison	
							Trigger	Symptom	LOS (d)	Outcome	Trigger	Symptom	LOS (*d*)	Outcome	Symptom	Imaging
1	38	F	21.48	Asthma	Non	8	URI	Dyspnea, cough	9	Improved	URI	Dyspnea, cough	8	Improved	Lighter	Lighter
2	25	M	15.37	Silicosis	Non	6.5	URI	Dyspnea	4	Improved	None	Dyspnea, chest pain	9	Improved	More severe	More severe
3	16	M	22.77	None	Non	13	None	Dyspnea, cough	OP	Improved	None	Dyspnea, cough	3	Improved	More severe	More severe
4	81	F	18.75	Bronchitis	Non	1	None	Dyspnea, cough	16	Improved	None	Dyspnea, cough	10	Recovered	More severe	More severe
5	56	M	15.57	PTB	Yes	16.5	URI	Dyspnea, cough	6	Improved	URI	Dyspnea, cough	16	Improved	Lighter	Lighter
6	13	M	16.67	None	Non	12.5	None	Chest pain	3	Improved	None	Chest pain	OP	Improved	Lighter	Lighter
7	14	M	17.78	Diabetes	Non	6	DKA	Dyspnea, nausea	7	Improved	DKA	Chest pain, nausea	5	Recovered	More severe	More severe
8	16	M	19.71	None	Yes	12	None	Dyspnea, chest pain, chest tightness	4	Recovered	Physical exertion	Chest pain	3	Improved	Lighter	Lighter
9	58	M	20.55	Silicosis, bronchitis	Non	1	URI	Dyspnea, cough, chest pain	10	Improved	URI	Dyspnea, cough, chest pain	21	Improved	Lighter	Lighter

*Note:* OP: outpatient treatment (not hospitalized). Non, nonsmoker; DKA, diabetic ketoacidosis; PTB, pulmonary tuberculosis; LOS, length of hospital stay; OP, outpatient.

Abbreviations: BMI, body mass index; *F*, female; *M*, male; URI, upper respiratory infection; *y*, years.

Dyspnea was the most common symptom at recurrence (66.7%), followed by cough (55.6%) and chest pain (55.6%). In 44.4% of the patients, no specific trigger was identified. Importantly, four patients (44.4%) exhibited more severe clinical symptoms and radiological findings at recurrence compared with their initial episode, while the other five showed milder manifestations. Regardless of severity, all patients improved or recovered with conservative management.

Univariate analysis comparing patients with and without recurrence is summarized in Table 4. Several factors showed a statistically significant association with recurrence: a history of silicosis (OR = 6.91, 95% CI: 1.13–42.17, *p* = 0.04), bronchitis (OR = 11.71, 95% CI: 1.68–81.89, *p* = 0.01), concurrent pneumonia (OR = 5.41, 95% CI: 1.28–22.86, *p* = 0.02), upper respiratory tract infection as a trigger (OR = 4.80, 95% CI: 1.18–19.59, *p* = 0.03), and the symptom of dyspnea (OR = 9.08, 95% CI: 1.1–74.79, *p* = 0.04). Cough showed a borderline association (OR = 3.67, 95% CI: 0.93–14.52, *p* = 0.06).

**TABLE 4:** Univariate analysis for predictors of recurrent pneumomediastinum.

Variables	Univariate analysis		
	OR	95% CI	*p* value
MH			
Silicosis	6.91	1.13–42.17	0.04
Bronchitis	11.71	1.68–81.89	0.01
Complication			
Pneumonia	5.41	1.28–22.86	0.02
Triggering events			
Upper respiratory infection	4.80	1.18–19.59	0.03
Symptoms			
Dyspnea	9.08	1.1–74.79	0.04
Cough	3.67	0.93–14.52	0.06

*Note:* PTB, pulmonary tuberculosis.

Abbreviations: CI, confidence interval; MH, medical history; OR, odds ratio.

## 4. Discussion

In this study, we summarized the characteristics of 135 patients with SPM. SPM occurs mostly in young men. Most patients had no clear triggers, and their symptoms were mainly chest pain and dyspnea. The most common sign was subcutaneous emphysema of the neck. These characteristics are consistent with those reported in previous studies [12, 13]. SPM is defined as an event in which free gas appears in the mediastinum without any obvious cause (such as chest trauma) [3]. Clinicians should recognize these typical features to avoid unnecessary invasive investigations in patients with uncomplicated SPM. These observations are consistent with previous larger series and can be considered confirmatory.

SPM is a benign, self‐limiting disease, and its management should focus on solving potential causes (if present), whereas patients with simple SPM do not require special intervention, mainly observation [18, 26]. In our cohort, the majority of patients with uncomplicated SPM were managed conservatively with oxygen and analgesia, with only two requiring closed thoracic drainage for pneumothorax. Rogers et al. have reported [19] that oxygen inhalation can increase the diffusion pressure of nitrogen in the stroma and promote the absorption of free air in the mediastinum, and analgesic treatment can effectively alleviate the symptoms of chest and neck pain, reduce the pain of patients, and play an active role in the control of shortness of breath. After treatment, all patients showed a benign clinical process, with a median hospital stay of 5 [11, 24] days, no serious complications, or death.

The trigger factors associated with SPM are recorded in many case series, including acute attacks of bronchial asthma, multiple movements, DKA, vomiting, childbirth, cough, and drug use. Among these, asthma has been described as one of the most commonly reported factors in 8%–39% of the cases [12–16]. In our study, the most common trigger factor was an upper respiratory tract infection (16.3%). This may be because some of the cases in this series that triggered SPM due to asthma attacks induced by upper respiratory tract infection were attributed to upper respiratory tract infection itself, which leads to a higher proportion of upper respiratory tract infections as trigger factors.

Viral respiratory infections (including COVID‐19 and influenza) have been reported as potential triggers of SPM through mechanisms such as severe coughing, alveolar inflammation, or increased intrathoracic pressure. In our cohort, upper respiratory tract infection was the most common identified trigger (16.3%). However, we cannot determine what proportion of these were due to specific viruses, as routine PCR testing for SARS‐CoV‐2 and influenza was not performed in all patients during the study period. Importantly, no patient in our series had a documented positive test for COVID‐19 or influenza at the time of SPM diagnosis. Nevertheless, given the retrospective design and incomplete testing, we cannot exclude the possibility that some patients with undetected viral infections were included. Future prospective studies should systematically investigate the role of emerging respiratory viruses in SPM.

The most classic sign of SPM is Hamman’s sign, which is a bubble burst sound synchronized with the heartbeat. This sound can be heard at the apex of the heart and the lower end of the sternum and is related to the occurrence and development of pneumomediastinum. However, this classic sign is not always observed, with positivity rates ranging from 0%–100% [17]. In our series, no patient had documented Hamman’s sign. This is because, with current medical developments, the diagnosis of this disease depends more on imaging diagnosis, and this positive rate is less stable. The signs were not taken seriously and were recorded. At the same time, it also reflects the necessity of studying and reporting the clinical characteristics of SPM, a rare disease.

In addition, we found that the incidence of SPM peaked in both the 15–19 years age group and the 50–54 years group. In view of this feature, we analyzed and compared the clinical characteristics of SPM in the adolescent (< 18 years) and the older (≥ 50 years) groups. These age cutoffs correspond to two physiologically distinct periods, adolescence and older adulthood, which informed the following comparisons. Compared with the older group, the incidence of SPM in the adolescent group was not easy to detect as an obvious trigger factor, and it was more often manifested as chest and neck pain, and the occurrence of hyperuricemia was more often observed. The incidence in older patients is more often associated with underlying lung diseases such as COPD and pneumonia and is mainly characterized by shortness of breath, coughing, and higher overall neutrophil levels. The distinct clinical profiles between the adolescent (< 18 years) and older (≥ 50 years) groups likely reflect different underlying pathophysiological mechanisms. In adolescents, the Macklin effect may be more frequently triggered by activities that generate high intra‐alveolar pressure without underlying lung disease (e.g., strenuous exercise and Valsalva maneuvers). In contrast, older patients often have pre‐existing lung conditions (COPD, silicosis, and bronchitis) and infections, which reduce the threshold for alveolar rupture. These age‐related differences support the development of age‐specific preventive strategies, e.g., avoiding extreme physical exertion in susceptible adolescents and optimizing control of chronic lung diseases and respiratory infections in older adults. In addition, the significantly elevated uric acid level in the young group is an interesting finding. Fujikawa et al. [10] showed that in patients with COPD, for every 1 mg/dL increase in serum uric acid, FEV1% decreased by 1.87% (95% CI: −2.91, −0.84), FVC % decreased by 1.35% (95% CI: −2.35, −0.34), and FEV1/FVC decreased by 0.63% (95% CI: −1.18, −0.08). While the clinical differences between adolescents and older adults are consistent with the proposed mechanisms, this interpretation remains hypothetical. The adolescent group’s lack of comorbidities and higher prevalence of physical exertion suggests a predominantly pressure‐driven mechanism, whereas the older group’s comorbidities and infections suggest a mechanism involving both structural vulnerability and exacerbation‐related pressure surges. However, these are post hoc pathophysiological inferences, and they should be tested in future mechanistic studies.

The SPM recurrence rate in this study was 6.7%, which is higher than the 0%–4.5% SPM recurrence rates reported in previous studies [20–25]. Because many RSPM symptoms are mild and have certain self‐limitations, many patients do not visit a doctor after the occurrence of related symptoms, resulting in an underestimation of the actual incidence. Recurrent pneumomediastinum was more common in men (77.8%). Most patients had underlying lung diseases (55.6%) or diseases highly associated with the incidence of SPM, such as diabetes (11.1%). Recurrence can occur 1–16.5 months after the first attack and often has the same inducement as that of the first attack. In contrast to the symptoms of SPM, recurrent pneumomediastinum manifests less frequently as chest pain, whereas shortness of breath and cough are more common.

Some previous studies [4, 18] have suggested that SPM recurrence is rare and, when it occurs, presents with similar or less severe clinical manifestations compared with the initial episode. Based on these findings, they proposed that the risk of recurrence may not warrant special concern and that hospitalization may not be necessary for recurrent episodes. However, in our case series, recurrent SPM was not always milder than the initial attack. A considerable proportion of patients (44.4%) exhibited greater severity in both symptoms and imaging findings at the time of recurrence. Additionally, 22.2% of the patients, despite presenting with less severe symptoms than at first admission, required a longer hospital stay to achieve symptom relief. In our univariate analysis, a history of bronchitis showed a strong association with RSPM (OR = 11.71). This finding differs from that of Yu et al. [5], who identified asthma as an independent risk factor in a larger cohort (237 cases). The discrepancy may be due to differences in the sample size or population characteristics. The wide CIs observed in our univariate analysis underscore the imprecision of the estimates, and the small event number (*n* = 9) limits the reliability of any strong conclusions regarding risk factors. These findings should serve primarily to inform the design of future larger studies. Therefore, for patients with underlying pulmonary diseases and complications, especially those with bronchitis or asthma, it is prudent to inform them about the possible risk of recurrence after the first SPM and to encourage timely medical consultation if symptoms recur.

Unlike SPM, which is usually managed conservatively, the disease progression and treatment of secondary pneumomediastinum are more aggressive. In a study of SPM and secondary PM cohorts by Magouliotis et al. [9], the secondary pneumomediastinum group showed remarkably higher morbidity, mortality, and LOS. However, SPM is usually difficult to distinguish from secondary pneumomediastinum in some subtle lesions, such as small esophageal perforations, slight tears of the central trachea or bronchial tree, and special mediastinal infections. Therefore, considering the risk of secondary pneumomediastinum, excluding pathological events that cause secondary pneumomediastinum when diagnosing SPM is important. The differential diagnosis of SPM is primarily based on imaging findings. Chest radiography is the most convenient, fast, and inexpensive screening method for diagnosing SPM. However, approximately 30% of SPM cannot be diagnosed using chest radiography [7]. Chest CT can detect pneumomediastinum even in patients with a small amount of mediastinal air or the Macklin effect [8]. Therefore, chest CT scan is the gold standard of imaging. For patients with suspected primary esophageal or bronchial rupture, esophagography and bronchoscopy should be performed to avoid a missed diagnosis of secondary pneumomediastinum. In this study, imaging was used to evaluate efficacy before discharge, in addition to being used as a diagnostic tool. When all patients complained of improved symptoms, different manifestations of complete absorption, partial absorption, and even aggravation of pneumomediastinum were still observed on imaging. This approach allows a more objective assessment of the patient’s condition compared to relying solely on symptoms, guiding further decision‐making.

Several limitations should be acknowledged. First, the single‐center retrospective design may introduce selection bias. Our institution is a tertiary referral center, which may have led to the inclusion of more severe or complicated cases, while milder cases managed in primary care or not seeking medical attention may have been underrepresented. Additionally, the retrospective nature of data collection may have resulted in incomplete documentation of trigger factors, symptoms, and imaging findings. These factors may limit the generalizability of our findings to broader or community‐based populations. Second, our use of the broad definition of SPM (including cases with underlying lung disease) may limit comparability with studies using stricter definitions. Third, and most importantly, the number of recurrent cases was very small (*n* = 9), which precluded multivariate analysis and resulted in wide CIs (e.g., for bronchitis: 95% CI: 1.68–81.89). Consequently, our findings regarding risk factors for RSPM are exploratory and hypothesis‐generating, not definitive. Large‐scale prospective studies are needed to validate these associations. Fourth, the follow‐up was passive and based on routine clinical visits rather than scheduled imaging surveillance. Consequently, some mild or asymptomatic recurrences may have been missed, potentially leading to underestimation of the true recurrence rate. In addition, the observation window was not uniform across the cohort: patients diagnosed after September 2020 had shorter potential follow‐up than those diagnosed earlier, which may have affected the comparability of recurrence estimates.

## 5. Conclusions

This large cohort study confirms that SPM predominantly affects young males and generally has a benign, self‐limiting course. A bimodal age distribution (peaks at 15–19 and 50–54 years) was observed, with distinct clinical profiles between adolescents and older adults. Although SPM recurrence is uncommon (6.7%), it may be more severe than the initial episode in nearly half of the cases. Univariate analysis suggested that a history of bronchitis and several other factors may be associated with recurrence, but these findings are exploratory due to the small number of recurrent events (*n* = 9). Clinicians should inform patients with underlying lung diseases about the possibility of recurrence and emphasize timely symptom monitoring. Age‐specific preventive strategies and careful exclusion of secondary pneumomediastinum using chest CT are essential.

**Nomenclature**
SPM: Spontaneous pneumomediastinum
RSPM: Recurrent spontaneous pneumomediastinum
OR: Odds ratio
CI: Confidence interval
HRCT: High‐resolution computed tomography
SD: Standard deviation
IQR: Interquartile range
ICD: International Classification of Diseases
MH: Medical history
PTB: Pulmonary tuberculosis
COPD: Chronic obstructive pulmonary disease
PB: Pulmonary bulla
DKA: Diabetic ketoacidosis
WBC: White blood cell
BMI: Body mass index
LOS: Length of hospital stay
URI: Upper respiratory infection
OP: Outpatient

## Author Contributions

(I) Conception and design: Chengbin Huang and Zhiyang Xu; (II) administrative support: Jun Guan; (III) provision of study materials or patients: Junkai Xiong; (IV) collection and assembly of data: Chengbin Huang, Zhizhou Chen, and Sheng Ouyang; (V) data analysis and interpretation: Chengbin Huang and Jianyang Wu; (VI) manuscript writing: all authors.

## Funding

The authors have nothing to report.

## Disclosure

All authors gave final approval for the manuscript.

## Ethics Statement

This study was approved by the Ethics Committee of The First Hospital of Putian City, which waived the requirement for informed consent due to the retrospective, noninterventional design. The study complied with the Declaration of Helsinki.

## Conflicts of Interest

The authors declare no conflicts of interest.

## Data Availability

The data that support the findings of this study are available from The First Hospital of Putian City but restrictions apply to the availability of these data, which were used under license for the current study, and so are not publicly available. Data are, however, available from the authors upon reasonable request and with permission of The First Hospital of Putian City.

## References

[1] Patel A. , Kesler B. , and Wise R. A. , Persistent Pneumomediastinum in Interstitial Fibrosis Associated With Rheumatoid Arthritis: Treatment With High-Concentration Oxygen, Chest. (June 2000) 117, no. 6, 1809–1813, 10.1378/chest.117.6.1809. 10858424

[2] Takada K. , Matsumoto S. , Hiramatsu T. et al., Spontaneous Pneumomediastinum: An Algorithm for Diagnosis and Management, Therapeutic Advances in Respiratory Disease. (2009) 3, no. 6, 301–307, 10.1177/1753465809350888. 19934282

[3] Hamman L. , Spontaneous Mediastinal Emphysema, Bulletin of the Johns Hopkins Hospital. (1939) 64, 1–21.

[4] Kumeda H. , Saito G. , Eguchi T. , Hara D. , and Shimizu K. , Clinical Features of Recurrent Spontaneous Pneumomediastinum, Journal of Thoracic Disease. (February 2023) 15, no. 2, 462–471, 10.21037/jtd-22-1141. PMC9992629 36910106

[5] Yu M. H. , Kim J. K. , Kim T. , Lee H. S. , and Kim D. K. , Primary Spontaneous Pneumomediastinum: 237 Cases in a Single-Center Experience Over a 10-year Period and Assessment of Factors Related With Recurrence, PLoS One. (July 2023) 18, no. 7, 10.1371/journal.pone.0289225. PMC10370696 37494372

[6] Talwar A. , Rajeev A. , Rachapudi S. , Khan S. , and Singh V. , Spontaneous Pneumomediastinum: A Comprehensive Review of Diagnosis and Management, Intractable Rare Dis Res. (August 2024) 13, no. 3, 138–147, 10.5582/irdr.2024.01020. PMC11350202 39220281

[7] Okada M. , Adachi H. , Shibuya Y. , Ishikawa S. , and Hamabe Y. , Diagnosis and Treatment of Patients With Spontaneous Pneumomediastinum, Respiratory Investigation. (2014) 52, no. 1, 36–40, 10.1016/j.resinv.2013.06.001. 24388369

[8] Sakai M. , Murayama S. , Gibo M. , Akamine T. , and Nagata O. , Frequent Cause of the Macklin Effect in Spontaneous Pneumomediastinum: Demonstration by Multidetector-Row Computed Tomography, Journal of Computer Assisted Tomography. (2006) 30, no. 1, 92–94, 10.1097/01.rct.0000187416.07698.8d. 16365580

[9] Magouliotis D. E. , Sgantzou I. , Salemis N. S. et al., Pneumomediastinum: Experience With 87 Patients, Acta Medica Academica. (August 2023) 52, no. 2, 88–94, 10.5644/ama2006-124.408. PMC10698360 37933505

[10] Fujikawa H. , Sakamoto Y. , Masuda N. et al., Higher Blood Uric Acid in Female Humans and Mice as a Protective Factor Against Pathophysiological Decline of Lung Function, Antioxidants. (2020) 9, no. 5, 10.3390/antiox9050387. PMC7278835 32384764

[11] Macklin M. T. and Macklin C. C. , Malignant Interstitial Emphysema of the Lungs and Mediastinum as an Important Occult Complication in Many Respiratory Diseases and Other Conditions: An Interpretation of the Clinical Literature in the Light of Laboratory Experiment, Medicine. (1944) 23, no. 4, 281–358, 10.1097/00005792-194412000-00001.

[12] Koullias G. J. , Korkolis D. P. , Wang X. J. , and Hammond G. L. , Current Assessment and Management of Spontaneous Pneumomediastinum: Experience in 24 Adult Patients, European Journal of Cardio-Thoracic Surgery. (2004) 25, 852–855, 10.1016/j.ejcts.2004.01.042. 15082293

[13] Newcomb A. E. and Clarke C. P. , Spontaneous Pneumomediastinum. A Benign Curiosity or a Significant Problem?, Chest. (2005) 128, 3298–3302. 10.1378/chest.128.5.3298 16304275

[14] Maunder R. J. , Pierson D. J. , and Hudson L. D. , Subcutaneous and Mediastinal Emphysema. Pathophysiology, Diagnosis, and Management, Archives of Internal Medicine. (July 1984) 144, no. 7, 1447–1453. 6375617

[15] Campillo A. , Coll A. , Soria V. et al., Neumomediastino Esponta ´Neo: Estudio Descriptivo de Nuestra Experiencia Basada en 36 Casos, Archivos de Bronconeumología. (2005) 41, 528–531. 10.1016/S1579-2129(06)60274-7 PMC7129626 16194517

[16] Jougon J. B. , Ballester M. , Delcambre F. , Mac Bride T. , Dromer C. E. H. , and Velly J. F. , Assessment of Spontaneous Pneumomediastinum: Experience With 12 Patients, Ann Thorac Surg. (2003) 75, no. 6, 1711–1714, 10.1016/s0003-4975(03)00027-4. 12822604

[17] Macia I. , Moya J. , Ramos R. et al., Spontaneous Pneumomediastinum: 41 Cases, European Journal of Cardio-Thoracic Surgery. (June 2007) 31, no. 6, 1110–1114, 10.1016/j.ejcts.2007.03.008. 17420139

[18] Wald L. , Yergin C. , Petroze R. , Larson S. , and Islam S. , The Unnecessary Workups and Admissions of Adolescents and Young Adults With Spontaneous Pneumomediastinum, Scientific Reports. (February 2024) 14, no. 1, 10.1038/s41598-024-55134-1. PMC10894201 38402248

[19] Rogers L. F. , Puig A. W. , Dooley B. N. , and Cuello L. , Diagnostic Considerations in Mediastinal Emphysema: A Pathophysiologic-Roentgenologic Approach to Boerhaave’s Syndrome and Spontaneous Pneumomediastinum, American Journal of Roentgenology, Radium Therapy & Nuclear Medicine. (1972) 115, no. 3, 495–511, 10.2214/ajr.115.3.495. 5038631

[20] Ebina M. , Inoue A. , Takaba A. , and Ariyoshi K. , Management of Spontaneous Pneumomediastinum: are Hospitalization and Prophylactic Antibiotics Needed?, The American Journal of Emergency Medicine. (2017) 35, no. 8, 1150–1153, 10.1016/j.ajem.2017.03.017. 28330688

[21] Bakhos C. T. , Pupovac S. S. , Ata A. , Fantauzzi J. P. , and Fabian T. , Spontaneous Pneumomediastinum: an Extensive Workup is not Required, Journal of the American College of Surgeons. (October 2014) 219, no. 4, 713–717, 10.1016/j.jamcollsurg.2014.06.001. 25053221

[22] Abolnik I. , Lossos I. S. , and Breuer R. , Spontaneous Pneumomediastinum: Areport of25 Cases, Chest. (1991) 100, 93–95. 10.1378/chest.100.1.93 1824034

[23] Gerazounis M. , Athanassiadi K. , Kalantzi N. , and Moustardas M. , Spontaneous Pneumomediastinum: A Rare Benign Entity, The Journal of Thoracic and Cardiovascular Surgery. (2003) 126, no. 3, 774–776, 10.1016/s0022-5223(03)00124-7. 14502153

[24] Macia I. , Moya J. , Ramo S. R. et al., Spontaneouspneumomed Iastinum: 41 Cases, European Journal of Cardio-Thoracic Surgery. (2007) 31, 1110–1114, 10.1016/j.ejcts.2007.03.008. 17420139

[25] Okada M. , Adachi H. , Shibuya Y. , Ishikawa S. , and Hamabe Y. , Diagnosis and Treatment Ofpatients With Spontaneous Pneumomediastinum, Respiratory Investigation. (2014) 52, no. 1, 36–40, 10.1016/j.resinv.2013.06.001. 24388369

[26] Iyer V. N. , Joshi A. Y. , and Ryu J. H. , Spontaneous Pneumomediastinum: Analysis of 62 Consecutive Adult Patients, Mayo Clinic Proceedings. (May 2009) 84, no. 5, 417–421, 10.1016/S0025-6196(11)60560-0. PMC2676124 19411438

